# Evaluation of a Community Health Worker Social Prescribing Program Among UK Patients With Type 2 Diabetes

**DOI:** 10.1001/jamanetworkopen.2021.26236

**Published:** 2021-09-01

**Authors:** John Wildman, Josephine M. Wildman

**Affiliations:** 1 Population Health Sciences Institute, Newcastle University, Newcastle upon Tyne, United Kingdom

## Abstract

**Importance:**

Despite lacking robust evidence of effectiveness, health care systems in developed countries are funding holistic community health worker (CHW) social prescribing programs that address social needs and health behaviors as adjuncts to clinical care.

**Objective:**

To determine whether a UK National Health Service (NHS) CHW social prescribing program was associated with improved hemoglobin A_1c_ (HbA_1c_) levels among patients with type 2 diabetes.

**Design, Setting, and Participants:**

This cohort study with difference-in-differences analysis was conducted among 8086 patients (4752 in the referral program, 3334 in the control group) in 24 NHS primary care practices in a city in North East England. Patients aged 40 to 74 years with a diagnosis of type 2 diabetes were observed for 8 years, from 2011 through 2019. The statistical analysis was conducted between June 1, 2019, and January 31, 2021.

**Interventions:**

A social prescribing program, launched in April 2015, enabling primary care staff to refer patients to CHW support to identify condition management and social needs goals and access voluntary and community sector support to address these goals. Intervention referral was only available in primary care practices in the city’s west. The control group included patients in the city’s east where referral was unavailable.

**Main Outcomes and Measures:**

HbA_1c_ level, a marker of glycemic control.

**Results:**

A total of 8086 patients were included in the analysis (mean [SD] age, 57.8 [8.78] years; 3477 women [43%]; 6631 White patients [82%]). Mean (SD) baseline HbA_1c_ levels were 7.56% (1.47%) in the referral program group and 7.44% (1.43%) in the control group. Following introduction of the social prescribing referral program, the referral group experienced an HbA_1c_ reduction of −0.10 percentage points (95% CI, −0.17 to −0.03 percentage points) compared with the control group. The association increased over time: after 3 years the estimated association was −0.14 percentage points (95% CI, −0.24 to −0.03 percentage points). The association was stronger for White patients compared with non-White patients (−0.15 percentage points [95% CI, −0.26 to −0.04 percentage points] after 3 years), those with fewer additional comorbidities (−0.16 percentage points [95% CI, −0.29 to −0.04 percentage points] after 3 years), and those living in the most socioeconomically deprived areas (−0.19 percentage points [95% CI, −0.32 to −0.07 percentage points] after 3 years).

**Conclusions and Relevance:**

In this study of UK adults with type 2 diabetes, a social prescribing program with referral to CHWs targeting patients’ social needs and health behaviors was associated with improved HbA_1c_ levels, suggesting that holistic CHW interventions may help to reduce the public health burden of type 2 diabetes.

## Key Points

**Question:**

Is a community health worker social prescribing program associated with improved glycemic control among UK patients aged 40 to 74 years with type 2 diabetes?

**Findings:**

In this cohort study with difference-in-differences analysis of 8086 patients in the UK National Health System, a holistic community health worker intervention was associated with improvements in hemoglobin A_1c_ levels. The association increased over time and was greater for White patients vs non-White patients, those with fewer additional comorbidities, and those living in the most socioeconomically deprived areas.

**Meaning:**

The community health worker social prescribing program was associated with improved glycemic control, suggesting that these types of interventions may help to reduce the public health burden of type 2 diabetes.

## Teaser

This cohort study examines whether a UK National Health Service community health worker social prescribing program was associated with improved hemoglobin A_1c_ levels among patients with type 2 diabetes

## Introduction

Health care systems as diverse as those of the US and the UK are funding interventions that identify and address patients’ social needs as an adjunct to clinical care.^1,2,3,4,5^ Interventions to tackle social needs share similar characteristics: identification of patient need by a member of clinical staff (in the UK, this can be general practitioner physicians or practice nurses); screening and assessment of priority needs; and community health worker (CHW) support to access community resources and activities. Several states in the US are implementing Medicaid-managed models incentivizing clinicians to work with community organizations to address the social determinants of health.^2^ In the UK, the term *social prescribing* has been adopted to describe these holistic interventions. England’s publicly funded National Health Service (NHS) is currently introducing a program of social prescribing, enabling primary care teams to refer patients to a link worker*,* which is a type of CHW who facilitates access to sources of voluntary and community sector support.^6^

Despite enthusiastic adoption of holistic CHW interventions, there is little robust evidence of effectiveness.^4,5,7^ To our knowledge, this study is the first large-scale analysis of the association between a social prescribing CHW referral program and long-term condition management, represented by changes in glycemic control among patients with type 2 diabetes.^8^ Our study focused on patients with type 2 diabetes for 3 reasons: First, many CHW interventions target people with long-term conditions such as type 2 diabetes that place substantial costs on health care systems.^9^ Second, there is a socioeconomic gradient in type 2 diabetes prevalence and complication rates.^10,11^ The role of social factors in the etiology and management of type 2 diabetes is reflected in the American Diabetes Association Clinical Practice Guidelines, which recommend screening patients for socioeconomic risk factors, referring to sources of community support, and providing self-management support through CHWs.^12^ Third, a routinely-collected clinical outcome (glycated hemoglobin, HbA_1c_) is available, which does not rely on self-report or selection into a study.

A comprehensive review by Kim et al^13^ found that CHW-delivered, life-style focused diabetes education programs can deliver improvements in glycemic control.^14,15^ However, only 1 study reports on the impact of a holistic CHW intervention targeting social needs in addition to lifestyle factors.^16^ Carrasquillo and colleagues^16^ conducted a randomized clinical trial of 300 Latino outpatients aged 18 to 65 years receiving type 2 diabetes treatment at a Florida public hospital between July 2010 and October 2013. Participants were randomized to receive enhanced usual care or a 1-year CHW intervention consisting of support with health behavior change and social needs. Follow-up data from 215 participants found that intervention participation was associated with a −0.51% (95% CI, −0.94% to −0.08%) reduction in HbA_1c_ levels.

In this study, we used a difference-in-differences analysis, exploiting the introduction of a social prescribing CHW referral program in a geographically selected area, to examine whether the intervention was associated with improved blood glucose control among patients with type 2 diabetes living in an area of socioeconomic deprivation in North East England.

## Methods

Ethical approval for this study was obtained from the Proportionate Review Sub-committee of the London-Brent Research Ethics Committee. Written informed patient consent was not required for registry data. Patients who chose to opt out of participation in research were flagged in general practice registers, identified by their NHS numbers and excluded from any data extraction processes by the North of England Commissioning Support Unit who provided the study's pseudonymized data. This cohort study followed the Strengthening the Reporting of Observational Studies in Epidemiology (STROBE) reporting guideline.

### The Intervention

The intervention aimed to improve health-related behaviors and condition management, with a focus on addressing the social determinants of health through CHW-facilitated access to voluntary and community sector services. Intervention components are described in detail in the study protocol and in the eAppendix in the Supplement.^8^ Briefly, patients eligible for referral were aged 40 to 74 years (the population eligible for the NHS Health Check program),^17^ with 1 or more of the following chronic health conditions: diabetes types 1 and 2, chronic obstructive pulmonary disease, asthma, heart failure, coronary heart disease, epilepsy, and osteoporosis. Core components of the intervention were patient needs identification, goal setting, and support to access community resources. Patients were referred by a primary care practitioner to a link worker (CHW), who helped patients identify condition management and social needs goals across 8 domains, covering lifestyle, self care, symptom management, work and volunteering, money, living conditions, social relationships, and mental well-being. Patients were supported to access appropriate services and community groups, such as welfare rights, employment support, and housing advice, in addition to health and lifestyle support. Participants could remain within the intervention for up to 2 years (or longer with link worker discretion). The intervention was personalized, with no typical composition or specified treatment. The nature and duration of patient and link worker interaction varied from frequent and intense to occasional and brief, depending on a patient’s needs and preferences. Reflecting the personalized goal setting, the nature and number of onward referrals to sources of community support was also heterogeneous.^18,19^

Since April 2015, the referral program has been offered to patients in 16 primary care practices in west Newcastle upon Tyne, an inner-city area ranked among England’s 40 most socioeconomically deprived areas. Around 1500 patients with type 2 diabetes had been referred into the program by March 31, 2019.

### Data and Study Population

The primary outcome was glycated hemoglobin (HbA_1c_) percentage, a marker of diabetic glycemic control, which provides a measure of mean plasma glucose over the preceding 8 to 12 weeks. The referral group comprised eligible patients in primary care practices where the social prescribing program was available. Referring practices were all located in Newcastle’s inner and outer west. The control group was eligible patients registered in practices in the city’s east (an area with similar levels of socioeconomic deprivation),^20^ where referral was not offered. Both treatment and control patients received usual care, which was the standard type 2 diabetes management.^21^

We used routinely collected NHS Quality and Outcomes Framework (QOF) data to measure individual patient HbA_1c_ (averaged across the year), sex, ethnicity, body mass index (BMI) (calculated as weight in kilograms divided by height in meters squared) and the presence of 1 or more additional comorbidity from the list of referral-qualifying conditions.^8^ Race and ethnicity data were self-reported by patients and follows the UK Census classifications of the following: British, Irish, other White; Black African, Black Caribbean, Black British, other Black, mixed Black; Bangladeshi, Pakistani, Indian, Sri Lankan, British Asian, other South Asian, mixed Asian, Chinese; other ethnic groups, other mixed groups. These categories are often grouped under the 4 main categories: White, Black, South Asian, and other. The QOF is an incentive program that financially rewards English general practices for quality of patient care. From QOF data, we also identified patients within the eligibility age range, registered at a treatment or control practice, having been diagnosed with type 2 diabetes on or before April 1, 2015. We did not include patients with type 1 diabetes. Permission to access QOF data was given by 13 of the 16 treatment practices, covering 80% of patients referred into the intervention, and 11 of the 15 control practices. Index of multiple deprivation (IMD) deciles were used as a measure of patients’ socioeconomic deprivation. The IMD is a measure of relative deprivation for small areas in England and ranked every lower super output area (LSOA, a geospatial statistical unit containing a mean of 1500 residents) from 1 (most deprived) to 32 844 (least deprived). IMD scores are grouped into deciles (eg, 1 to 3284 represented the 10% most deprived neighborhoods). Deciles were linked with LSOA data provided by the North of England Commissioning Support Unit. (Detailed variable definitions are given in eTable 1 in the Supplement.)

### Statistical Analysis

We estimated difference-in-differences 2-way (individual and time) fixed-effects models to compare changes in outcomes between individuals in referral practices vs control practices before and after the introduction of the referral program on April 1, 2015. The 2-way fixed-effects model was conducted to help control for time and unobserved individual factors that may confound the association between treatment (ie, referral) and the outcome.

As we were analyzing data from a natural experiment, assignment to the referral treatment program group or control group was not under researcher control. Furthermore, the intervention, and natural experiments more broadly, were characterized by heterogeneity in intervention composition, compliance, and dose.^22^ Therefore, we estimated an intention-to-treat (ITT) analysis, acknowledging that this provided a conservative estimate of any association between the referral program and the outcome, and that our results represented treatment assignment and not treatment received.^23^

All models included a quadratic in age. As we were conducting an ITT analysis, we took the conservative route of applying standard errors clustered at the general practice level (results with standard errors clustered at the individual level are available in eTable 2 and 3 in the Supplement). We used yearly data from April 1, 2011 (4 years before the program launched) until March 31, 2019 (4 years after the program launched), giving a sample size for the overall model of 49 752 observations, an unbalanced panel for 8086 individuals (4752 individuals in the referral group and 3334 individuals in the control group) (eFigure in the Supplement shows the number of patients appearing in each year). Individuals, on average, appeared in 6 waves of the data.

To examine within-group associations, we conducted subgroup analyses based on pretreatment baseline characteristics, by sex, age group (aged 55 years and under or over 55 years), racial and ethnic group, presence of obesity (BMI greater than or equal to 30), presence of comorbidities (no additional morbidity, 1 extra, and 2 or more extra), and area-level socioeconomic deprivation deciles. For analysis, individuals were grouped into White and non-White categories. More than 80% of the sample identified as White; thus, there were insufficient numbers to analyze non-White racial and ethnic categories separately. To investigate the validity of our assumptions, we conducted analysis using lead and lag periods as a falsification test. Analysis of treatment and control groups matched on observable characteristics, using inverse probability weights, was conducted as a robustness check. All hypothesis tests were 2-sided and 95% CIs were calculated with *P* < .05 considered to be statistically significant. Statistical analyses were conducted using Stata software version 16.0 (StataCorp) from June 1, 2019, to January 31, 2021.

## Results

A total of 8086 patients were included in the analysis (mean [SD] age, 57.8 [8.78] years; 3477 women [43%]; 6631 White [82%]). Of these, 6797 (84%) were observed both before and after program implementation. Baseline mean (SD) HbA_1c_ levels were 7.56% (1.47%) in the referral program group and 7.44% (1.43%) in the control group. Data for the referral and control groups are presented in Table 1.

**Table 1.** Summary Statistics for Included Participants^a^

Characteristic	Participants, No. (%)			Difference, percentage points	*P* value
	Total	Treatment	Control		
HbA_1c_, mean (SD), %^b^	7.51 (1.46)	7.56 (1.47)	7.44 (1.43)	0.12	<.001
Age, mean (SD), y	57.77 (8.78)	57.59 (8.86)	58.02 (8.65)	−0.43	<.001
Sex					
Women	8773 (43)	5145 (43)	3628 (42)	1	.14
Men	11 825 (57)	6815 (57)	5010 (58)		
Comorbidity					
No additional	8712 (42)	5316 (44)	3396 (39)	5	<.001
Additional	11 886 (58)	6644 (56)	5242 (61)		
Race					
Non-White^c^	3683 (18)	2768 (23)	915 (11)	12	<.001
White^d^	16 765 (82)	9087 (77)	7678 (89)		
Socioeconomic level					
Most deprived^e^	11 783 (57)	7024 (59)	4759 (55)	4	<.001
Least deprived	8788 (43)	4910 (41)	3878 (45)		

Abbreviation: HbA_1c_, hemoglobin A_1c_.

^a^ Summary statistics based on pretreatment characteristics for an unbalanced panel of 8086 individuals. Means and counts are presented for 20 598 (N × T) observations (treated = 11 960; control = 8638), apart from ethnicity (n = 20 448, *t* = 11 855, C = 8593) and most deprived (n = 20 571, *t* = 8637, C = 11 934).

^b^ HbA_1c_ values are reported as Diabetes Control and Complications Trial units (%). To convert to International Federation of Clinical Chemistry units (mmol/mol), multiply by 10.93 and subtract 23.5.

^c^ Non-White: Black African, Black Caribbean, Black British, other Black, mixed Black, Bangladeshi, Pakistani, Indian, Sri Lankan, British Asian, other South Asian, mixed Asian, Chinese, other ethnic groups, other mixed groups.

^d^ White: British, Irish, other White.

^e^ Patients living in the 2 most deprived index of multiple deprivation (IMD) deciles.

**Figure 1.**
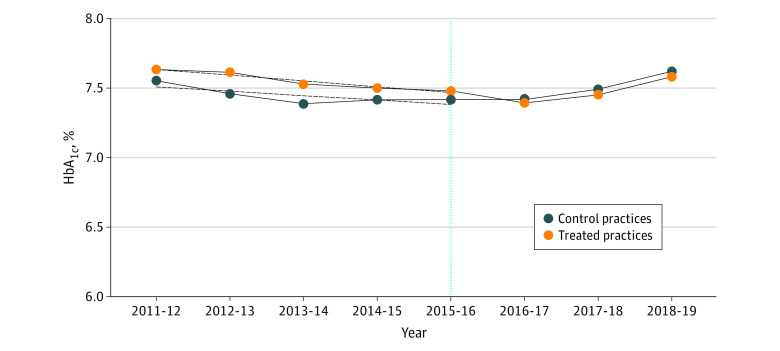
Mean HbA_1c_ by Year The figure shows the unconditional mean HbA_1c_ over time for the 2 groups, with the fitted linear time trend. The yellow dots and blue dots are joined to form trend lines—one for the control practices (blue dots) and one for treated practices (yellow dots). The solid lines are the lines of best fit in the period before treatment. The vertical line indicates the year of intervention implementation. HbA_1c_ indicates hemoglobin A_1c_. Toconvert Diabetes Control and Complications Trial units (%) to International Federation of Clinical Chemistry units (mmol/mol), multiply by 10.93 and subtract 23.5.

Figure 1 shows the unconditional HbA_1c_ trends from years 2011-2012 to 2018-2019. Changes in HbA_1c_ associated with the referral program, represented by marginal effect sizes, are given in Table 2 and Table 3. Table 1 shows that before the referral program, compared with the control group, the referral group had higher baseline mean (SD) HbA_1c_ levels (referral: 7.56% (1.47%) vs control: 7.44% [1.43%]; difference, 0.12 percentage points; *P* < .001), were more likely to have no additional comorbidities (referral: 5316 individuals [44%] vs control: 3396 individuals (39%); difference, 5 percentage points; *P* < .001) and live in areas of greater deprivation (referral: 7024 individuals [59%] vs control: 4759 individuals [55%]; difference, 4 percentage points; *P* < .001), were younger (mean [SD] age in referral group: 57.59 [8.86] years vs control group: 58.02 [8.65] years; difference, −0.43 years; *P* < .001), and were more likely to be non-White individuals (referral: 2768 individuals [23%] vs control: 915 individuals [11%]; difference, 13 percentage points; *P* < .001).

**Table 2.** Difference-in-Differences Results After Program Launch: Part A^a^^,^^b^

Variable	Difference-in-differences for HbA_1c_ (95% CI), percentage points^c^					Constant	N × T^d^	*R* ^2^	F
	Treated	2015-2016	2016-2017	2017-2018	2018-2019				
Overall model	−0.10 (−0.17 to −0.03)	NA	NA	NA	NA	20.68 (−2.99 to 44.35)	49 752	0.65	19.00
By years	NA	−0.05 (−0.10 to 0.01)	−0.10 (−0.15 to −0.05)	−0.12 (−0.23 to −0.01)	−0.14 (−0.24 to −0.03)	20.80 (−2.87 to 44.47)	49 752	0.65	12.86
Men	NA	−0.05 (−0.13 to 0.03)	−0.07 (−0.13 to −0.004)	−0.10 (−0.23 to 0.04)	−0.14 (−0.26 to −0.02)	25.62 (−3.75 to 55.00)	28 399	0.63	3.95
Women	NA	−0.04 (−0.13 to 0.05)	−0.14 (−0.24 to −0.05)	−0.15 (−0.26 to −0.05)^I^	−0.14 (−0.24 to −0.03)	0.67 (−40.97 to 42.30)	21 353	0.68	20.26
White^e^	NA	−0.05 (−0.12 to 0.01)	−0.11 (−0.17 to −0.05)	−0.11 (−0.24 to 0.02)	−0.15 (−0.26 to −0.04)	12.88 (−5.01 to 30.76)	40 299	0.64	8.91
Non-White^e^	NA	0.003 (−0.10 to 0.10)	−0.04 (−0.16 to 0.09)	−0.11 (−0.27 to 0.04)	0.01 (−0.16 to 0.17)	52.37 (−31.47 to 136.22)	9045	0.69	4.99
Age ≤55 y	NA	−0.005 (−0.09 to 0.08)	−0.09 (−0.19 to 0.02)	−0.11 (−0.29 to 0.06)	−0.14 (−0.28 to −0.001)	7.13 (−10.64 to 24.90)	23 752	0.65	3.13
Age >55 y	NA	−0.08 (−0.16 to −0.004)	−0.11 (−0.16 to −0.07)	−0.13 (−0.20 to −0.06)	−0.13 (−0.22 to −0.04)	55.16 (14.42 to 95.90)	26 000	0.62	11.01

Abbreviations: HbA1c, hemoglobin A1c; NA, not applicable.

^a^ Standard errors were clustered at practice level.

^b^ Fixed-effects models for individuals were estimated. Fixed effects models control for all time invariant observable and unobservable characteristics. All models include time dummies and a quadratic for age.

^c^ To convert these estimates from Diabetes Control and Complications Trial (%) to International Federation of Clinical Chemistry (mmol/mol), multiply by 10.93.

^d^ Sample sizes are N × T for an unbalanced panel.

^e^ White categories: British, Irish, other White. Non-White categories: Black African, Black Caribbean, Black British, other Black, mixed Black, Bangladeshi, Pakistani, Indian, Sri Lankan, British Asian, other South Asian, mixed Asian, Chinese, other ethnic groups, other mixed groups.

**Table 3.** Difference-in-Differences Results After Program Launch: Part B^a^^,^^b^

Variable	Difference-in-differences for HbA_1c_ (95% CI), percentage points^c^				Constant (95% CI)	N × T^d^	*R* ^2^	F
	2015-2016	2016-2017	2017-2018	2018-2019				
No comorbidity	−0.07 (−0.16 to 0.03)	−0.14 (−0.22 to −0.05)	−0.17 (−0.31 to −0.04)	−0.11 (−0.23 to 0.004)	12.03 (−17.31 to 41.37)	21 495	0.65	4.82
1 comorbidity	−0.06 (−0.14 to 0.01)	−0.13 (−0.21 to −0.04)	−0.13 (−0.29 to 0.03)	−0.16 (−0.29 to −0.04)	33.68 (−11.77 to 79.14)	17 263	0.65	10.05
≥2 comorbidities	0.02 (−0.07 to 0.11)	0.02 (−0.13 to 0.16)	−0.01 (−0.21 to 0.20)	−0.13 (−0.34 to 0.07)	2.174 (−1.42 to 5.76)	10 994	0.65	16.82
No obesity	−0.09 (−0.15 to −0.03)	−0.12 (−0.18 to −0.04)	−0.14 (−0.27 to −0.01)	−0.16 (−0.31 to −0.01)	24.88 (−19.02 to 68.77)	28 671	0.64	20.87
Obesity	0.01 (−0.08 to 0.10)	−0.08 (−0.18 to 0.03)	−0.09 (−0.22 to 0.04)	−0.11 (−0.21 to −0.01)	15.96 (8.06 to 23.85)	20 995	0.66	9.15
Most deprived	−0.05 (−0.15 to 0.05)	−0.12 (−0.19 to −0.04)	−0.20 (−0.31 to −0.08)	−0.19 (−0.32 to −0.07)	5.95 (−17.18 to 29.09)	16 856	0.67	4.39
Least deprived	−0.05 (−0.11 to 0.02)	−0.10 (−0.16 to −0.03)	−0.084 (−0.21 to 0.04)	−0.11 (−0.23 to 0.01)	30.13 (−6.51 to 66.78)	32 896	0.64	7.72

^a^ Standard errors were clustered at practice level.

^b^ Fixed-effects models for individuals were estimated. Fixed-effects models controlled for all time invariant observable and unobservable characteristics. All models included time dummies and a quadratic for age.

^c^ To convert these estimates from Diabetes Control and Complications Trial (%) to International Federation of Clinical Chemistry (mmol/mol), multiply by 10.93.

^d^ Sample sizes were N × T for an unbalanced panel.

A key identifying assumption for difference-in-differences is the untestable parallel trends assumption for the treatment and control groups, before treatment. Figure 1 shows the unconditional mean HbA_1c_ over time for the 2 groups, with the fitted linear time trend. The vertical line indicates the year of intervention implementation. Before the referral program, mean HbA_1c_ levels in both groups followed a similar parallel trend, providing support for our identifying assumptions. However, these are unconditional means, which could be confounded by other factors, such as age and sex. For this reason, it is important to consider the regression results and the results from the falsification test.

**Figure 2.**
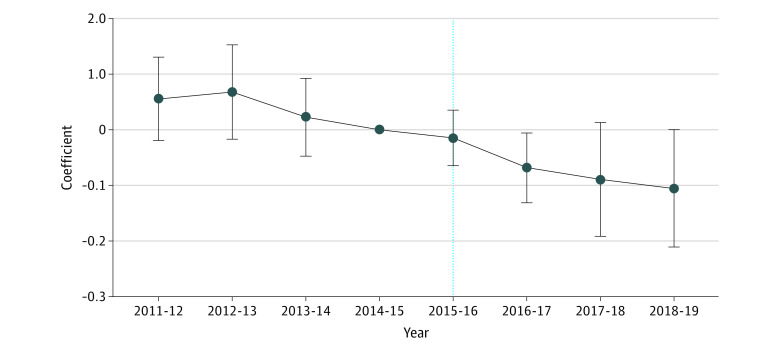
Plot of Leads and Lags Coefficients Estimates of yearly difference in HbA_1c_ (%) between the treated and the control groups, before and after program implementation. Error bars denote 95% CIs.

Table 2 shows that treatment was associated with a statistically significant reduction in HbA_1c_ (−0.10 percentage points [95% CI, −0.17 to −0.03 percentage points]). This model was also estimated on a matched sample, using inverse probability weights, for characteristics before the intervention, and the results were similar (reported in eTable 4, eTable 5, and eTable 6 in the Supplement). Table 2 shows how the association evolved across the years. HbA_1c_ levels decreased for the referral group relative to the control group. Two years after treatment, the estimated association was −0.12 percentage points (95% CI, −0.23 to −0.01 percentage points); after 3 years, it was −0.14 percentage points (95% CI, −0.24 to −0.03 percentage points). Leads and lags of the association from the falsification test are plotted in Figure 2. The estimates, which are conditional on age and the observable and unobservable time-invariant factors, show the conditional difference in mean HbA_1c_ levels between referral group and control group in each period, with 2014-2015, the year prior to program implementation, as the omitted year. Prior to program implementation, estimates were positive and mostly insignificant. In the period following the launch of the program, estimates were negative and, in the case of 2016, significant: the estimate for 2016 was −0.07 percentage points (95% CI, −0.13 to 0.00), showing that HbA_1c_ levels had decreased in the referral group relative to the control group after launch of the program.

Also in Table 2, separate estimates by sex, race and ethnicity, and age are presented. Sex, race and ethnicity, and age demonstrated similar patterns, with HbA_1c_ levels decreasing over time for the referral group, although the estimate for 2018 to 2019 was positive and close to 0 for non-White individuals. The analysis showed that the association was stronger for White patients compared with non-White patients (−0.15 percentage points [95% CI, −0.26 to −0.04 percentage points] after 3 years). Table 3 gives estimates by the presence of extra comorbidities, obesity status before the program, and socioeconomic deprivation. The results showed that the association was stronger for those with fewer additional comorbidities (−0.16 percentage points [95% CI, −0.29 to −0.04 percentage points] after 3 years), no obesity (−0.16 percentage points [95% CI, −0.31 to −0.01 percentage points] after 3 years) and those living in the most socioeconomically deprived areas (−0.19 percentage points [95% CI, −0.32 to −0.07 percentage points] after 3 years).

## Discussion

In this cohort study using difference-in-differences analysis, we found that improved glycemic control was associated with a holistic CHW-delivered, social prescribing program targeting adults aged 40 to 74 year with a diagnosis of type 2 diabetes living in an area of high socioeconomic deprivation in North East England. Our findings support those of a US study reporting a reduction in HbA_1c_ levels among Latino patients participating in a CHW intervention addressing health behaviors and the social determinants of health.^16^

While the estimates are small in absolute size with limited clinical significance, our findings suggest that self care and condition management appear to have improved in the group eligible for referral into the CHW social prescribing. The results are encouraging for holistic CHW interventions for 3 reasons. First, we conducted an intention-to-treat analysis. The inclusion of all eligible patients, regardless of whether they participated, is likely to attenuate the size of the estimates. Second, CHW interventions are not directly targeted at reducing HbA_1c_ (or any other clinical outcome). The management of HbA_1c_ is complex and Barnard et al^10^ have proposed a logic model conceptualizing the well-documented links between socioeconomic deprivation and type 2 diabetes complications: material insecurity reduces access to resources such as appropriate diet, medication, and exercise opportunities, while also increasing stress and reducing the capacity for self-efficacy. A cycle may occur of worsening symptoms, exacerbating stress and distress and diminishing the capacity for change.^10^ A holistic CHW intervention aims to break this cycle by linking participants with community resources with the intention of addressing the social needs associated with self care and condition management. As a result, improvements in glycemic control are likely to be modest compared with improvements achieved through a targeted pharmaceutical approach. Indeed, even an intensive lifestyle intervention directly targeting diabetes risk factors showed only modest reductions in HbA_1c_ compared with metformin.^24^ Third, the magnitude of the association with HbA_1c_ increased year-on-year compared with the control group. Qualitative research with participants in this program identified complex and interacting socioeconomic, physical, and mental health barriers to condition management. Although many participants were able to make lifestyle changes, setbacks were common and long-term support was often required.^18,19^ This highlights that, as noted by Kangovi et al^25^, interventions aimed at addressing social needs take time to yield results. The observed lag in improvement may also partly reflect that the longer the intervention is available, the more individuals can be referred into it.

Subgroup analyses shed light on whether the intervention was associated with better outcomes for some specific groups.^1^ In addition to type 2 diabetes, many participants receiving this intervention live with multiple chronic health conditions.^8^ The results for comorbidities suggest that individuals with less comorbidity (defined by intervention-qualifying comorbidities) may benefit more than individuals with 2 or more comorbidities. The burden of living with comorbidity is likely to reduce self-efficacy and the capacity to make behavioral changes.^26^ By 2018 to 2019, the size of the estimate for patients with multiple comorbidities was approaching the estimate size for those with fewer comorbidities, suggesting a capacity to benefit, albeit more slowly. The estimates for White patients were larger than those for racial and ethnic minority patients. A potential explanation is again offered by qualitative research in which CHWs reported a lack of local culturally-appropriate support services and activities to which they could refer racial and ethnic minority patients, limiting these patients potential to engage with the intervention and access the support they needed.^18^ Finally, there is a risk that an intervention will increase inequalities between socioeconomic groups and inequalities among patients with type 2 diabetes appear resistant to public health interventions.^1,11^ However, in this study, the association between the referral program and HbA_1c_ levels was stronger for individuals living in the most deprived areas (the 2 highest-ranked deprivation deciles).

One advantage of a difference-in-differences approach is that, under certain assumptions, it is possible to make causal inferences. There are 2 assumptions required for potential causal inference: First, the provision of the intervention in geographically selected general practices allowed us to consider referral to a CHW as an exogenous event: individuals cannot self-select into the referral program (patients tend to be registered at local general practices and switching practices is uncommon).^27^ There may be factors associated with general practices that affect engagement with the referral program; however, because none of individuals in this study changed general practice during the time period, our individual fixed effects analysis have absorbed general practice fixed effects controlling for unobservable time-invariant practice heterogeneity. The second assumption is that of unconfoundedness (ie, the referral program may not have coincided with other interventions that could vary between the 2 groups and that could influence HbA_1c_ levels). All practices were located within the same Clinical Commissioning Group (CCG), which purchase services for their local population in accordance with National Institute for Health and Care Excellence guidelines, meaning that usual type 2 diabetes care was likely to have been similar between referral and control groups. Our plots of parallel trends and the falsification test lend support to these assumptions (Figure 1 and Figure 2). However, as outlined in the limitations, we were not necessarily able to address all possible time varying unobserved confounders and report the findings as associations.

### Limitations

Our study has a number of limitations. Given the observational nature of this study, even with the difference-in differences analysis and the assumptions tested, the findings are at potential risk for unobserved confounding. It is the case that not all patients have a visit to their GP practice that results in the completion of QOF data in all years before and after program implementation, leading to a lack of balance between the groups across time. However, the use of fixed effects may help control for compositional changes in the preintervention and postintervention groups. Findings for racial and ethnic minority groups were limited by poor data recording and small group sizes. An ITT analysis cannot distinguish how much of the observed effect sizes were attributable to individual-level changes and how much were attributable to changes occurring at the general-practice level. This is an area for further research. Seven general practices refused permission for patient data to be included in the study, reducing the sample size. In addition, although all of the general practices in this study are part of the same CCG, providing some homogeneity of treatment, it is possible that, beyond usual care, some control practices were referring patients to other condition management interventions. If additional referrals were occurring during the period of our analysis, this would result in conservative estimates. Finally, the effect sizes and differences reported were statistically small and may not have clinical significance. However, glycemic control has the largest effect on health over the lifetime (compared with systolic blood pressure, total cholesterol and high density lipoprotein levels),^28^ and any reduction in HbA_1c_ levels are likely to be beneficial.^29,30,31^

## Conclusions

In this cohort study with a difference-in-differences analysis of UK adults with type 2 diabetes, a social prescribing program with referral to CHWs targeting patients’ social needs and health behavior was associated with improved HbA_1c_ levels. These findings suggest that social prescribing CHW-interventions addressing the wider social determinants of health may play a role in reducing the long-term public health burden of type 2 diabetes.

## Notes

Supplement. **eAppendix.** Intervention Description **eTable 1.** Variable Names And Definitions **eTable 2.** DiD Results, Part A With Standard Errors Clustered at The Individual Level **eTable 3.** DiD Results, Part B With Standard Errors Clustered at The Individual Level **eTable 4.** Summary Statistics With Inverse Probability Weights **eTable 5.** DiD Results, Part A With Inverse Probability Weights **eTable 6.** DiD Results, Part B With Inverse Probability Weights **eFigure.** Number of Patients Appearing in Each Year
