# Supplementary material for: Evaluation of a Community Health Worker Social Prescribing Program Among UK Patients With Type 2 Diabetes
Source: JAMA Netw Open. 2021 Sep 1;4(9):e2126236. doi: 10.1001/jamanetworkopen.2021.26236 (PMC8411296; doi:10.1001/jamanetworkopen.2021.26236)
Supplement: Supplement. — eAppendix. Intervention Description eTable 1. Variable Names And Definitions eTable 2. DiD Results, Part A With Standard Errors Clustered at The Individual Level eTable 3. DiD Results, Part B With Standard Errors Clustered at The Individual Level eTable 4. Summary Statistics With Inverse Probability Weights eTable 5. DiD Results, Part A With Inverse Probability Weights eTable 6. DiD Results, Part B With Inverse Probability Weights eFigure. Number of Patients Appearing in Each Year [file jamanetwopen-e2126236-s001.pdf]

Supplemental Online Content

Wildman J, Wildman JM. Evaluation of a Community health worker social prescribing program among UK patients with type 2 diabetes. *JAMA Netw Open.* 2021;4(9):e2126236. doi:10.1001/jamanetworkopen.2021.26236

eAppendix. Intervention Description

eTable 1. Variable Names And Definitions

eTable 2. DiD Results, Part A With Standard Errors Clustered at The Individual Level

eTable 3. DiD Results, Part B With Standard Errors Clustered at The Individual Level

eTable 4. Summary Statistics With Inverse Probability Weights

eTable 5. DiD Results, Part A With Inverse Probability Weights

eTable 6. DiD Results, Part B With Inverse Probability Weights

eFigure. Number of Patients Appearing in Each Year

This supplemental material has been provided by the authors to give readers additional information about their work.

eAppendix: Intervention description

The intervention is a 'social prescribing' intervention that enables primary care staff to refer patients aged 40 to 74 with a range of chronic health conditions to a 'link worker' (a type of community health worker). Approximately 40% of referred patients have a diagnosis of type 2 diabetes. The intervention has four stated aims: (1) to improve patients' health-related behaviours, (2) to improve self-care, (3) to encourage better long-term condition self-management, and (4) to promote social integration. There is a strong focus on addressing the wider determinants of health by supporting patients to access services such as welfare, debt and housing advice, and employment or volunteering opportunities. The intervention is delivered by not-for-profit organisations.

Patients who accept a referral are allocated a 'link worker' who has completed a training programme comprising an established Health Trainer National Vocational Qualifications, motivational interviewing, goal identification and setting, understanding chronic health physical and mental health conditions, and the availability of local support services. Link workers' employing organisations also hold data on local community and voluntary services. Beyond their formal training, link workers have identified their wide-ranging professional backgrounds in health training, welfare rights advice and support work as providing a valuable extra source of knowledge around local services. Goal identification, setting and progress monitoring is conducted using a tool that helps patients assess their state across eight parameters (lifestyle; self-care; symptom management; work, volunteering and activity; money; home environment; personal relationships; positive feeling). For each client, link workers provide the following: (a) individual assessment, motivational interviewing and action planning; (b) goal identification and planning at baseline (the initial patient/link worker appointment) and at 6-month intervals up to 24 months (or longer if required); (c) support and guidance to access community services (e.g. walking groups, physical activity classes and welfare rights advice); (d) promotion of volunteering opportunities; (e) promotion of improved self-care.

The intervention is highly personalised involving face-to-face contacts in community settings, accompanying patients as they attend services or activities, and, where necessary, domiciliary visits. Contact is also made, where appropriate, via email, text and telephone. All contacts vary in duration and frequency in accordance with individual client need. The intervention was deliberately designed to be highly flexible and patient-need driven and, for this reason, there is no typical 'dose'. The potential for replication of the intervention arises from its core processes. The elements that should be replicated are regular patient/community health worker contacts (over a period of months rather than weeks) involving facilitated identification of patient needs, goal setting, and support to access community and voluntary sector services.

© 2021 Wildman J et al. *JAMA Network Open.*

Variables description

eTable 1: Variable names and definitions

Variable	Definition
HbA1c (hemoglobin A1c)	Expressed as a percentage
Age	Individual age, years
Women	A dummy (0/1) indicating whether an individual's recorded sex in the QOF[a] is female (1) or male (0)
Co-morbidities	A categorical variable indicating:<br>0 – no additional morbidity<br>1 – one additional morbidity<br>2 – two or more additional morbidities<br>These values are taken from the QOF and are time invariant.
Ethnicity, non-white	A dummy (0/1) variable indicating whether an individual is ethnically non-white (1) or white (0).
Deprivation decile (Index of Multiple Deprivation (IMD), deciles).	The IMD is the official measure of relative deprivation for small areas in England and ranks every Lower Super Output Area (LSOA) (an LSOA is a geospatial statistical unit containing an average of 1500 residents) from 1 (most deprived) to 32844 (least deprived)). IMD scores are grouped into deciles (e.g. 1 to 3284 represents the 10% most deprived neighbourhoods). Deciles were linked with LSOA data provided by the North of England Commissioning Support Unit. Decile 1 (10) represents being in the 10% most (least) deprived LSOAs.<br><br>The IMD combines information from the seven domains to produce an overall relative measure of deprivation. The domains are combined using the following weights:<br>•Income Deprivation (22.5%)<br>•Employment Deprivation (22.5%)<br>•Education, Skills and Training Deprivation (13.5%)<br>•Health Deprivation and Disability (13.5%)<br>•Crime (9.3%)<br>•Barriers to Housing and Services (9.3%)<br>•Living Environment Deprivation (9.3%)<br><br>LSOA location, and so IMD decile, is taken from the QOF and is time invariant.

Abbreviation: QOF, Quality and Outcomes Framework
[a] Quality and Outcomes Framework

© 2021 Wildman J et al. *JAMA Network Open.*

Estimated treatment effects with individual-level standard errors

eTable 2: DiD results, Part A with standard errors clustered at the individual level[a,b]

	Treated	2015/16	2016/17	2017/18	2018/19	Constant	N*T[c]	R^2	F
(1) Overall model	-0.102[e]					20.68	49752	0.649	18.42
	[-0.150,-0.0529]					[-4.208,45.56]			
(2) By years		-0.0460[d]	-0.100[f]	-0.121[f]	-0.137[f]	20.80	49752	0.649	9.764
		[-0.0988,0.00667]	[-0.158,-0.0416]	[-0.190,-0.0520]	[-0.200,-0.0741]	[-4.137,45.73]			
(3) Men		-0.0486	-0.0662	-0.0951[e]	-0.137[f]	25.62[d]	28399	0.626	3.824
		[-0.120,0.0230]	[-0.146,0.0137]	[-0.188,-0.00243]	[-0.221,-0.0521]	[-2.484,53.73]			
(4) Women		-0.0415	-0.144[f]	-0.154[f]	-0.137[f]	0.666	21353	0.680	7.522
		[-0.119,0.0361]	[-0.229,-0.0586]	[-0.257,-0.0511]	[-0.231,-0.0428]	[-37.60,38.94]			
(5) Ethnicity White		-0.0515[d]	-0.106[f]	-0.114[f]	-0.146[f]	12.88	40299	0.639	7.464
		[-0.109,0.00657]	[-0.170,-0.0422]	[-0.190,-0.0373]	[-0.215,-0.0777]	[-7.952,33.71]			
(6) Ethnicity non-white		0.00306	-0.0392	-0.113	0.00784	52.37	9045	0.692	3.726
		[-0.140,0.146]	[-0.198,0.120]	[-0.292,0.0661]	[-0.161,0.177]	[-23.85,128.6]			
(7) 55 years or under		-0.00470	-0.0858[d]	-0.113[e]	-0.143[f]	7.128	23752	0.653	3.162
		[-0.0916,0.0822]	[-0.181,0.00898]	[-0.225,-0.000773]	[-0.246,-0.0395]	[-13.54,27.79]			
(8) Over 55 years		-0.0825[e]	-0.112[f]	-0.127[f]	-0.132[f]	55.16[e]	26000	0.623	3.446
		[-0.146,-0.0188]	[-0.185,-0.0402]	[-0.211,-0.0428]	[-0.208,-0.0565]	[0.355,110.0]			

[a] 95% confidence intervals in brackets
[b] Standard errors were clustered at individual level. Fixed effects models for individuals were estimated. Fixed effects models control for all time invariant observable and unobservable characteristics. All models include time dummies and a quadratic for age.
[c] Sample sizes are N*T for an unbalanced panel
[d]$p < 0.10$, [e]$p < 0.05$, [f]$p < 0.01$
To convert estimates from DCCT (%) to IFFC (mmol/mol) multiply the estimates by 10.93

© 2021 Wildman J et al. *JAMA Network Open.*

eTable 3: DiD results, Part B with standard errors clustered at the individual level[a,b]

	2015/16	2016/17	2017/18	2018/19	Constant	N*T[c]	R^2	F
(1) No co-morbidity	-0.0658	-0.137[f]	-0.174[f]	-0.114[f]	12.03	21495	0.649	4.496
	[-0.145,0.014]	[-0.225,-0.049]	[-0.279,-0.070]	[-0.208,-0.019]	[-15.349,39.405]			
(2) One co-morbidity	-0.0625	-0.125[e]	-0.133[e]	-0.164[f]	33.68	17263	0.653	5.382
	[-0.152,0.027]	[-0.224,-0.026]	[-0.252,-0.014]	[-0.270,-0.058]	[-10.219,77.587]			
(3) Two+ co-morbidities	0.0219	0.0158	-0.00578	-0.134[d]	2.174	10994	0.646	5.974
	[-0.094,0.137]	[-0.111,0.143]	[-0.150,0.138]	[-0.271,0.004]	[-1.094,5.443]			
(4) Not obese	-0.0888[e]	-0.115[f]	-0.140[f]	-0.158[f]	24.88	28671	0.643	6.616
	[-0.162,-0.015]	[-0.194,-0.035]	[-0.235,-0.045]	[-0.246,-0.071]	[-16.923,66.678]			
(5) Obese	0.00784	-0.0772[d]	-0.0934[d]	-0.109[e]	15.96	20995	0.659	3.933
	[-0.067,0.083]	[-0.163,0.009]	[-0.193,0.007]	[-0.200,-0.019]	[-4.328,36.240]			
(6) Most deprived	-0.0515	-0.115[e]	-0.197[f]	-0.194[f]	5.951	16856	0.668	4.018
	[-0.135,0.032]	[-0.213,-0.016]	[-0.307,-0.088]	[-0.296,-0.092]	[-15.667,27.570]			
(7) Least deprived	-0.0458	-0.0972[f]	-0.0840[d]	-0.107[f]	30.13	32896	0.640	6.819
	[-0.112,0.021]	[-0.170,-0.025]	[-0.172,0.003]	[-0.186,-0.029]	[-5.853,66.120]			

[a] 95% confidence intervals in brackets
[b] Standard errors were clustered at individual level. Fixed effects models for individuals were estimated. Fixed effects models control for all time invariant observable and unobservable characteristics. All models include time dummies and a quadratic for age.
[c] Sample sizes are N*T for an unbalanced panel
[d] $p < 0.10$, [e] $p < 0.05$, [f] $p < 0.01$
To convert estimates from DCCT (%) to IFFC (mmol/mol) multiply the estimates by 10.93

© 2021 Wildman J et al. *JAMA Network Open.*

Inverse probability weights results.

Inverse probability weights were estimated, based on pre-treatment characteristics (age prior to treatment, sex, multi-morbidities, ethnicity and deprivation), for the sample with available data for all variables considered.

The summary statistics for the weighted sample are:

eTable 4: Summary statistics with inverse probability weights

	mean	SD	min	max	count
**Control**					
Hba1c, (%)	7.45	1.43	4.16	13.13	8592
Age, years	57·89	8·73	36	73	8592
Women	0·43	0·50	0	1	8592
Multi morbidities	0·42	0·49	0	1	8592
Ethnic, non-white	0·18	0·38	0	1	8592
Deprivation [a]	0·57	0·50	0	1	8592
**Treatment**					
Hba1c, (%)	7.53	1.47	4.16	13.13	11829
Age, years	57·77	8·77	36	73	11829
Women	0·43	0·49	0	1	11829
Multi morbidities	0·42	0·49	0	1	11829
Ethnic, non-white	0·18	0·38	0	1	11829
Deprivation [a]	0·57	0·50	0	1	11829
**Total**					
Hba1c, (%)	7.49	1.45	4.16	13.13	20421
Age, years	57·83	8·75	36	73	20421
Women	0·43	0·50	0	1	20421
Multi morbidities	0·42	0·49	0	1	20421
Ethnic, non-white	0·18	0·38	0	1	20421
Deprivation [a]	0·57	0·50	0	1	20421

Abbreviation: HbA1c, hemoglobin A1c
[a] Patients living in the two most deprived IMD deciles

© 2021 Wildman J et al. *JAMA Network Open.*

eTable 5: DiD results, Part A with inverse probability weights[a,b]

	Treated	2015/16	2016/17	2017/18	2018/19	Constant	N*T[c]	R^2	F
(1) Overall model	-0.0922[e]					33.91[d]	49258	0.650	40.41
	[-0.158,-0.027]					[-1.115,68.939]			
(2) By years		-0.0422	-0.0951[f]	-0.116[e]	-0.115[e]	34.05[d]	49258	0.650	22.77
		[-0.099,0.014]	[-0.145,-0.045]	[-0.210,-0.021]	[-0.219,-0.010]	[-0.959,69.061]			
(3) Men		-0.0439	-0.0699[e]	-0.0890	-0.129[e]	40.46[e]	28081	0.627	6.728
		[-0.135,0.047]	[-0.131,-0.009]	[-0.220,0.042]	[-0.242,-0.015]	[3.259,77.665]			
(4) Women		-0.0391	-0.128[f]	-0.150[f]	-0.0964[d]	-3.273	21177	0.679	16.94
		[-0.136,0.058]	[-0.219,-0.037]	[-0.247,-0.052]	[-0.209,0.017]	[-45.583,39.037]			
(5) Ethnicity White		-0.0489	-0.102[f]	-0.111[d]	-0.140[e]	15.15[d]	40238	0.640	9.139
		[-0.114,0.016]	[-0.162,-0.041]	[-0.241,0.020]	[-0.248,-0.033]	[-1.334,31.632]			
(6) Ethnicity non-white		-0.0106	-0.0668	-0.135	-0.00374	83.47[f]	9020	0.685	5.129
		[-0.124,0.103]	[-0.194,0.060]	[-0.307,0.036]	[-0.185,0.177]	[28.303,138.629]			
(7) 55 years or under		0.0104	-0.0780	-0.102	-0.109	10.45	23489	0.653	1.951
		[-0.079,0.100]	[-0.185,0.029]	[-0.259,0.055]	[-0.245,0.026]	[-6.959,27.856]			
(8) Over 55 years		-0.0874[e]	-0.110[f]	-0.126[f]	-0.118[e]	77.44[f]	25769	0.625	14.74
		[-0.168,-0.007]	[-0.158,-0.062]	[-0.197,-0.054]	[-0.215,-0.020]	[40.192,114.688]			

[a] 95% confidence intervals in brackets
[b] Standard errors were clustered at individual level. Fixed effects models for individuals were estimated. Fixed effects models control for all time invariant observable and unobservable characteristics. All models include time dummies and a quadratic for age.
[c] Sample sizes are N*T for an unbalanced panel
[d] $p < 0.10$, [e] $p < 0.05$, [f] $p < 0.01$
To convert estimates from DCCT (%) to IFFC (mmol/mol) multiply the estimates by 10.93

© 2021 Wildman J et al. JAMA Network Open.

eTable 6: DiD results, Part B with inverse probability weights[a,b]

	2015/16	2016/17	2017/18	2018/19	Constant	N*T[c]	R^2	F
(1) No co-morbidity	-0.0561	-0.140[f]	-0.179[f]	-0.0909	16.84	21230	0·649	5·022
	[-0.148,0.036]	[-0.219,-0.061]	[-0.305,-0.053]	[-0.205,0.023]	[-13.271,46.942]			
(2) One co-morbidity	-0.0803[e]	-0.127[f]	-0.115	-0.161[e]	52.82[d]	17132	0·653	10·25
	[-0.160,-0.000]	[-0.219,-0.036]	[-0.278,0.048]	[-0.299,-0.022]	[-8.541,114.189]			
(3) Two+ co-morbidities	0.0445	0.0431	0.0104	-0.0932	1.332	10896	0·647	22·85
	[-0.044,0.133]	[-0.095,0.181]	[-0.183,0.204]	[-0.288,0.102]	[-2.324,4.989]			
(4) Not obese	-0.0886[f]	-0.117[f]	-0.138[e]	-0.148[d]	46.96[d]	28451	0·643	37·64
	[-0.148,-0.029]	[-0.181,-0.052]	[-0.261,-0.016]	[-0.296,0.001]	[-9.382,103.292]			
(5) Obese	0.0206	-0.0660	-0.0849	-0.0713	16.24[f]	20807	0·660	9·750
	[-0.070,0.111]	[-0.170,0.038]	[-0.191,0.021]	[-0.163,0.020]	[10.159,22.312]			
(6) Most deprived	-0.0556	-0.107[f]	-0.175[f]	-0.147[e]	6.175	16659	0·669	5·410
	[-0.160,0.049]	[-0.184,-0.030]	[-0.268,-0.082]	[-0.266,-0.027]	[-17.498,29.847]			
(7) Least deprived	-0.0370	-0.0916[f]	-0.0865	-0.0984[d]	46.43[e]	32599	0·640	10·88
	[-0.102,0.028]	[-0.152,-0.031]	[-0.207,0.034]	[-0.216,0.019]	[0.069,92.785]			

[a] 95% confidence intervals in brackets
[b] Standard errors were clustered at individual level. Fixed effects models for individuals were estimated. Fixed effects models control for all time invariant observable and unobservable characteristics. All models include time dummies and a quadratic for age.
[c] Sample sizes are N*T for an unbalanced panel
[d] $p < 0.10$, [e] $p < 0.05$, [f] $p < 0.01$
To convert estimates from DCCT (%) to IFFC (mmol/mol) multiply the estimates by 10.93

© 2021 Wildman J et al. JAMA Network Open.

Patient numbers in each year

eFigure: Number of patients appearing in each year

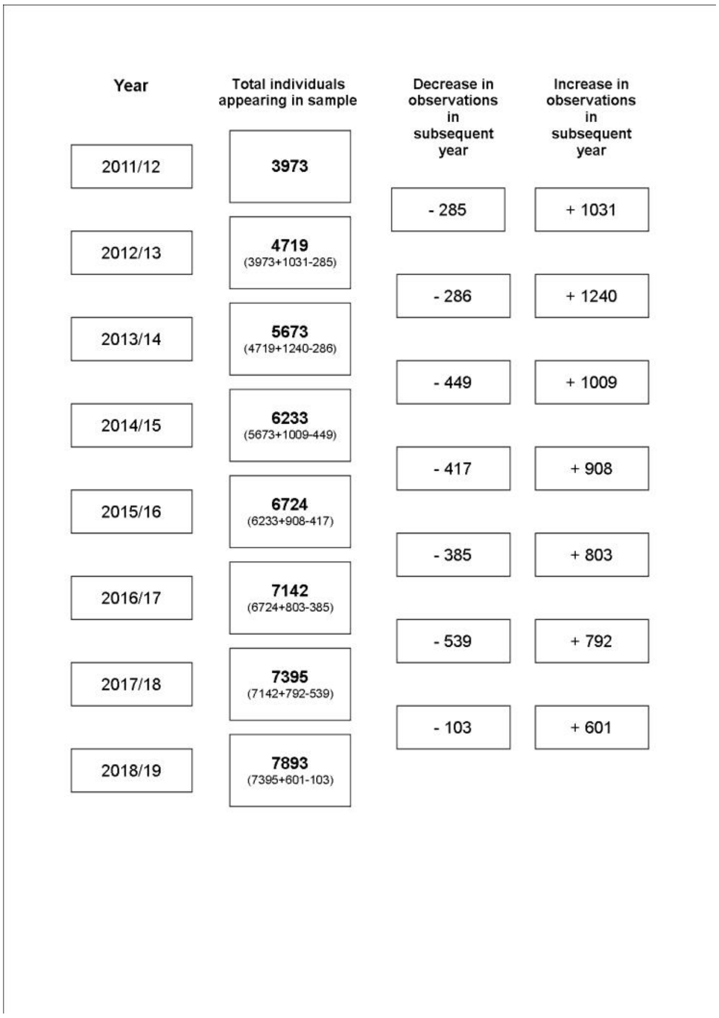

© 2021 Wildman J et al. JAMA Network Open.
